# Sexual and reproductive health communication and awareness of contraceptive methods among secondary school female students, northern Ethiopia: a cross-sectional study

**DOI:** 10.1186/1471-2458-14-252

**Published:** 2014-03-14

**Authors:** Yohannes Adama Melaku, Yemane Berhane, John Kinsman, Hailemariam Lemma Reda

**Affiliations:** 1 Department of public Health, College of Health Sciences, Mekelle University, Mekelle, Ethiopia; 2 Department of Epidemiology and biostatistics, Addis continental Institute of Public Health, Addis Ababa, Ethiopia; 3 Umeå Centre for Global Health Research, Department of Public Health and Clinical Medicine, Umeå University, Umeå 901 85, Sweden

**Keywords:** School students, Parent communication, Peer communication, Sexual and reproductive health, Contraceptive methods, Mekelle

## Abstract

**Background:**

Adolescent girls continue to fall victim to unintended pregnancy and its consequences, with particular problems arising in low income countries. Awareness about methods of contraception is an important step towards gaining access and using suitable contraceptive methods. However, studies assessing the relationship between sexual and reproductive health communication and awareness of contraceptive methods among secondary school female students are lacking.

**Methods:**

A cross sectional study was conducted among 807 female students in six secondary schools in Mekelle town, Ethiopia. Study participants were selected with a stratified cluster sampling technique. Data collection was carried out using a structured, self-administered questionnaire, and data entry was done using EPI Info Version 3.3.2 software. The data were then cleaned and analyzed using SPSS version 20. Bivariate and multivariate logistic regressions were used to determine factors associated with awareness of female students on methods of contraception.

**Result:**

Of all the students, 127(15.8%) reported ever having had sex, of whom 109(85.8%) had ever used contraceptives. Twenty (16%) of the sexually active students reported having been pregnant, of whom 18(90%) terminated their pregnancies with induced abortion. Discussion on sexual and reproductive health matters with their parent/s and peer/s in the six months prior to the study was reported by 351(43.5%) and 493(61.1%) of the students respectively. 716(88%) students were aware of different methods of contraception. Discussing sexual and reproductive health issues with parents (AOR =2.56(95% CI: 1.45, 4.50)) and peers (AOR = 2.46(95% CI: 1.50, 4.03)) were found to be independent predictors for contraceptive awareness among students.

**Conclusions:**

Discussion on sexual and reproductive health issues with family and peers has a positive effect on contraceptive awareness of students. Therefore, strategies to improve open parent–child communication, and appropriate peer-to-peer communication in schools on sexual and reproductive health should be established and strengthened.

## Background

Adolescence is defined as the second decade of life, the age between 10 and 19 years [1]. It is the time of transition from childhood to adulthood during which young people experience rapid physical, social and psychological changes as a result of puberty [2].

Young people aged between 15 and 24 years make up 1.2 billion of the world’s population [3]. The health threats faced by this group are predominantly behavioral, and they can have potentially serious consequences [4]. Since young people live in a life phase of experimentation and discovery, they are exposed to health-related risks such as unwanted pregnancies [5]. Thus the group deserves due attention with a special focus on sexual and reproductive behaviors [6].

It is estimated that 11% births globally are given by adolescent girls aged 15–19, and that 95% of these births take place in low income countries. Ethiopia is one of the countries with a high adolescent birth rate [7,8].

Each year there are about 250 million pregnancies globally; around one third of these are unintended, of which 20% end in induced abortion. Similar rates apply in low income countries, where more than one third of the 182 million pregnancies are unintended, of which 19% are subjected to abortion. However, 11% of these abortions are unsafe [9], with about 2 · 5 million (almost 14%) of all unsafe abortion in these countries occurring in women younger than 20 years. Unsafe abortion varies substantially by age across regions: 15–19 year olds account for 25% of all unsafe abortions in Africa, whereas the proportion in Asia, Latin America, and the Caribbean is much lower [10-12].

Adolescent women who become pregnant face a greater risk of pregnancy-induced health problems and complications during childbirth than women who bear their first child at age 20 and above [13-16]. Giving birth during adolescence is also associated with a higher than average rate of spontaneous and induced abortions [17], which can also affect the health of the mother. Not only does early child bearing have links to higher rates of maternal and child morbidity, but it can also lead to truncated educational opportunities, lower future family income, and large family size [17-20]. These can all have devastating impacts on the social, educational and psychological life of the girls [7].

Negative outcomes of early pregnancy and Sexually Transmitted Infections (STIs), including Human Immunodeficiency Virus/Acquired Immunodeficiency Syndrome (HIV/AIDS), threaten the health of people in the second decade of life more than any other age group [21]. Early sexual activity has clear negative academic consequences for girls when it results in pregnancy, and it predicts lower school performance and expectations for college among girls even in the absence of pregnancy [22].

Awareness about sexual and reproductive health (SRH) matters, specifically about methods of contraception, as an important step towards gaining access to, and using a suitable contraceptive method in a timely and effective manner. A lack of awareness about methods and use of contraception exposes adolescents to the risk of unwanted pregnancy, unsafe abortion, teenage delivery, child bearing, school dropout and various complications including death [4].

A cross sectional study among sexually active high school students in South Africa indicated that 48.8% of the males and 49.1% of females had used contraception. The most common contraceptive used by males was condom (81.4%), while among females it was the injection (65.4%). Only 17.8% of the males and 22.5% of females reported always using contraception [23]. A study in Uganda also showed that condoms and coitus interruptus were the most commonly used methods used by youths [24]. Among high school students in Nigeria, only 5% of 1155 who had knowledge of contraception were using any form of contraceptive methods. In the same study, 85% of the sexually active respondents were not using any form of contraception [25].

Analysis of the 2005 Demographic and Health Survey in Ethiopia showed that three quarters of the youth aged 15–24 years knew at least one contraceptive method. Awareness was found to be lower among unmarried youth who are not sexually experienced. Men are generally more aware of contraception than women, and knowledge varies by contraceptive method. Knowledge of modern methods of contraception was substantially higher than knowledge of traditional methods among both women and men. Nearly 60% of women were aware of injectable contraceptives compared with about 50% of men [26]. Another study, conducted in north Gondar found that 75% of those surveyed had knowledge of contraception [27].

Communication about sex between parents and children is potentially an important means of transmitting sexual values, beliefs, expectations, and knowledge [28]. However, inter-generational discussions on sex-related matters are taboo in much of Africa [29], with some adults believing that informing adolescents about sex and teaching them how to protect themselves would make them sexually active [30].

This also applies in Ethiopia, where parent-youth communication on SRH issues is believed to be culturally shameful [31]. In Zeway, Ethiopia, one study indicated that only 20% of parents had ever discussed SRH issues with their children [32]. In another study, 32% of young people (32.4% of females and 32.7% of males) had ever discussed SRH issues with their parents. In the same study, youths with secondary school and above educational status (AOR = 1.70, 95% CI: 1.30-2.24), as well as those in a younger age group (AOR = 1.57, 95% CI: 1.26-1.97) were more likely to engage in discussion with their parents compared to the less educated and older respondents [33].

In Ethiopia, studies conducted to explore the effect of parent and peer communication about SRH issues or contraceptive methods are rare. Studies that have been conducted, in Ethiopia and elsewhere in Africa, have concentrated on awareness of contraceptive utilization and emergency contraceptives, rather than awareness about specific methods of contraception [23-27].

The purpose of this study is, therefore, to determine the effect of parent and peers communication about SRH issues on awareness of contraceptive methods among secondary school female students in northern Ethiopia. It is hoped that the study will contribute to the design, implementation and evaluation of adolescent reproductive health programs, especially behavioral change communication programs, to enhance the reproductive health of young people. The findings will also act as baseline data for other researchers, policy makers and institutions working in this area.

## Methods

### Study design and setting

The study was conducted in Mekelle, the capital city of Tigray Region, located about 800 km north of Addis Ababa (capital city of Ethiopia). There were 25 secondary schools at the time of the study (11 public and 14 private), with more than 21,000 students. In the academic year 2011/2012, when the study was conducted, 10,056 female students were attending classes in the town’s secondary schools.

A cross sectional study among female students was conducted in six randomly selected secondary schools in Mekelle, of which three were publicly run and three were private.

### Sample size and sampling procedure

Sample size was determined using a single population proportion formula, with the following assumptions: maximum allowable error (5%); proportion of students having awareness of contraceptive methods (50%); Z statistic = 1.96; design effect of 2 (to compensate the clustering effect introduced as result of using stratified sampling technique); and non-response rate 10%. This gave a total sample size of 845. However, 38 female students were excluded during analysis because they were married, giving the final sample size of 807.

Proportional allocation for population size was used to determine the number of study participants in each school and in each grade level. Sections, which were considered as clusters, were selected in each grade level (grade 9th, 10th, 11^th^ and 12nd). All female students in the selected sections (clusters) were invited to participate in the study.

### Study variables

The outcome variable of the study was awareness on contraceptive methods, and the explanatory variables included were socio-demographic status; educational level of participants’ parents; sexual and reproductive health characteristics; media exposure; and reported discussion with peer and parent about sexual and reproductive health issues. In the analysis, categorization of variables was based on previous studies conducted in Ethiopia [8,18].

### Operational definition

**Awareness on contraceptive methods**-hearing/knowing about any methods of contraceptives, mentioning at least one specific type of method.

**Discussion with parent**-having talked about at least one sexual and reproductive health-related topic with their parent/s during the last six months.

**Discussion with peer**- having talked about at least one sexual and reproductive health-related topic with their peer/s during the last six months.

**Sexually active**- having a previous history of vaginal sexual intercourse.

**Sexual and reproductive health**-**related topics**-in this study this includes HIV/AIDS/STI, unwanted pregnancy and abortion, sexual organs and sexual intercourse, contraceptive methods and menstruation.

### Data collection tool and procedures

A pretested, self-administered, structured questionnaire (including both open-ended and closed–ended questions) was used in the study. The tool was mainly adapted from the Ethiopian Demographic and Health Survey (EDHS) data collection tool [8,18], which takes contextual issues into account. The English version of the structured questionnaire was translated into the local language, Tigrigna.

Data collection facilitators, who were female university students, were trained on the contents of the questionnaire, procedures for data collection, data accuracy and completeness. Field work was conducted under close supervision.

In each school, an official communication was used to identify a suitable time for data collection. Before completing the questionnaire, respondents were given a clear introduction explaining the purpose and objectives of the study. Respondents were similarly assured about the confidentiality and privacy of their responses. To avoid information contamination, data were collected during a single day in each participating school. Data collection took place in the absence of class teachers, and efforts were made to ensure maximum comfort and privacy for the participants. Students were sat apart from each other, and discussion was not allowed when completing the questionnaires, both for reasons for privacy and to avoid shared responses. When they had finished, students were requested to put their completed questionnaires into a sealed cartoon box instead of giving them to data collection facilitators.

### Data processing and statistical analysis

Data were edited, coded, cleaned for consistency, and entered into Epi Info 3.3.2 software. They were then exported to Statistical Package for Social Sciences (SPSS) version 20 software for analysis. The analysis aimed to determine the proportion of secondary school female students who had awareness about methods of preventing pregnancy: if a respondent knew at least one method of preventing pregnancy, she would be considered as being aware of contraceptive methods.

The association between the outcome variable (awareness on contraceptive methods) and several predictor variables (participants socio-demographic characteristics, sexual and reproductive health characteristics, media exposure, discussion with peer and/or parent about SRH issues, and participants’ parent educational level) were first analyzed in the bivariable logistic regression model. In the second step, predictor variables having p-value < 0.05 were retained and entered to the multivariate logistic regression analysis. A p-value < 0.05 was considered as a cut off point for a predicator to be significantly associated with the outcome. In the analyses, Hosmer-Lemeshow chi2 result was assessed to check goodness-of-fit of the models. As a result, all p-values were greater than 0.05 in the models we have developed.

### Ethical considerations

Ethical approval and an ethical clearance letter were obtained from the Institutional Review Board of Mekelle University. The Education offices of Mekelle town as well as school directors were informed through formal letters. Discussions were made with school directors regarding the purpose and the contents of the data collection tool, and permission was obtained. Respondents were given assurances about the privacy and confidentiality of their responses. Written consent was obtained from each participant to ensure their willingness to participate in the study, and they were told that all have a right to refuse to participate or to withdraw at any time. No data were given to any third party.

## Results

### Socio-demographic and sexual characteristics of respondents

A total of 807 study participants took part in the study, giving a response rate of 100%. The mean age of respondents’ was 16.55 years (standard deviation = 1.35), with a range from 13 to 21 years. The majority of the students, 685(84.9%), were living in an urban area before they joined secondary school. Most of them, 690(85.5%), were followers of Orthodox Christianity, and 695(86.1%) were attending their education in public schools. About 348(43%) of the study participants were grade 11 and 12 (senior classes), of whom 261(75.2%) were enrolled in the natural science stream (Table 1).

**Table 1:** **Socio**-**demographic and sexual characteristics of secondary school female students in Mekelle town**, **northern Ethiopia**, **2012**

**Characteristics**	**Frequency**	**Percent (%)**
**Age in years**		
13-15	188	23.3
16-17	397	49.2
18-21	222	27.5
**Mean** **±** **SD**	16.55 ± 1.35	
**Origin of residence**		
Rural	122	15.1
Urban	685	84.9
**Religion**		
Orthodox	690	85.5
Muslim	66	8.2
Other	51	6.3
**Educational level**		
9th grade	205	25.4
10th grade	254	31.5
11th grade	116	14.4
12th grade	232	28.7
**Stream of study (n = 348)**		
Social sciences	86	24.7
Natural sciences	262	75.3
**School**		
Public	695	86.1
Private	112	13.9
**Ever had sexual partner**		
Yes	171	21.2
No	636	78.8
**Do you have sexual partner currently? (n** = **171)**		
Yes	127	74.3
No	44	25.7
**Ever had sexual intercourse (n = 802)**		
Yes	127	15.8
No	675	84.2
**Ever had sexual intercourse in past 12 months (n** **=** **127)**		
Yes	80	63.0
No	47	37.0
**Ever had sexual intercourse in the past 3 months (n** **=** **80)**		
Yes	38	47.5
No	42	52.5
**Ever had pregnancy (N** **=** **127)**		
Yes	20	15.7
No	107	84.3
**History of induced abortion (n** **=** **20)**		
Yes	18	90
No	2	10

More than one fifth, 171(21.2%), of the participants ever had at least one sexual partner, of whom about three quarters, 127(74.3%), had a partner at the time of the study. Of those who had a sexual partner during the study period, 80(63.0%) had had sexual intercourse at least once in the past twelve months and 38(47.5%) at least once in the past three months prior to the study. Among sexually active respondents, 20(15.7%) had been pregnant at least once previously and 18(90%) of these young women had had an induced abortion (Table 1).

### Awareness and utilization of contraceptive methods

Seven hundred sixteen (88.7%) of the study participants reported that they had ever heard about ways of preventing pregnancy, mentioning at least one contraceptive method. The most commonly mentioned methods were the male condom (66.3%) and injectables (65.9%). The least known type of contraceptive by the students was withdrawal method (22.1%). The majority, 596(83.2%) of the students who knew about contraceptive methods knew at least one place where to obtain them (Table 2).

**Table 2:** **Awareness and utilization of specific type of contraceptive methods among secondary school female students**, **Mekelle town**, **northern Ethiopia**, **2012**

**Contraceptive methods**	**Known by students (N = 716)**	**Ever used by students (N = 109)**
	**Frequency (%)**	**Frequency (%)**
Male condom	475(66.3)	56(50.9)
Injectables	472(65.9)	3(2.7)
Oral pills	414(57.8)	
Implant	367(48.9)	
IUD	341(47.6)	7(6.9)
Emergency Oral Contraceptives (EOC)	318(39.4)	11(10.0)
Calendar method	265(37.0)	11(10.0)
Vasectomy	264(36.9)	
Tubal ligation	248(34.6)	
Diaphragm	201(28.1)	
LAM	193(27.0)	
Female condom	191(26.7)	
Withdrawal	158(22.1)	16(14.5)
Other methods	15(2.1)	

Five hundred sixty five (70%) of the students reported that abstinence is a method to prevent pregnancy. A large majority of the respondents, 717(88.8%), thought that both partners are responsible either to use or not to use contraceptive methods. Only a few reported that it is solely the male’s or the female’s responsibility (10.2% and 1.0% respectively).

Of all the participants, 318(39.4%) had awareness of the Emergency Oral Contraceptive (EOC) method. When asked about the maximum acceptable time to take it after sexual intercourse, 7(2.2%), 118(37.1%) and 77(24.2%), reported 120, 72, and 24 hours, respectively. Concerning the risk of pregnancy at first sexual intercourse, 66(8.2%) of the students reported that this was possible, while 350(48.9%) reported no risk of pregnancy, and 300(37.2%) were not sure.

Additionally, to assess students’ understanding of conception, the study assessed their awareness of the safest time during the menstrual cycle for a girl to have sexual intercourse: just before menses, during menses, or just after menses. Only 40(5.6%) respondents who had awareness on contraceptive methods had an accurate understanding of safest timing (just before, during, and just after menses).

Of those who reported having had sex (n = 127), 109(86.5%), had ever used some type of contraceptive method. The most commonly used type of contraceptive method was the condom, 56(50.9%), followed by the withdrawal method, 16(14.5%) (Table 2). Of those two contraceptive methods, about two-thirds, 82(75.2%), reportedly used them during their last sexual intercourse.

Respondents who were in the senior class (93.4%), or who had discussed SRH issues with either parent/s (94.9%) or peer/s (93.9%), were more likely to be aware of contraceptive methods than junior class students (85.2%), or those who had not discussed SRH issues with parents or peers (Table 3).

**Table 3:** **Discussion with parent**/**s and peer**/**s about SRH issues and awareness on contraceptive methods**, **Mekelle town**, **northern Ethiopia**, **2012**

**Characteristics**	**Had awareness**		**Pearson CHI2**
	**Yes**	**No**	**p-value**
**Age**			0.023
13-15 years	163(86.7%)	25(13.3%)	
16-17 years	345(86.9%)	52(13.1%)	
18-21 years	208(93.7%)	14(6.3%)	
**Educational level**			< 0.001
Junior	391(85.2%)	68(14.8%)	
Senior	325(93.4%)	23(6.6%)	
**Discussion with parent/s**			< 0.001
Yes	333(94.9%)	18(5.1%)	
No	383(84%)	73(16.0%)	
**Discussion with peer/s**			< 0.001
Yes	463(93.9%)	30(6.1%)	
No	253(80.6%)	61(19.4%)	
**Ever had sex (n** **= 802)**			< 0.001
Yes	125(98.4%)	2(1.6%)	
No	586(86.8%)	89(13.2%)	

### Sources of information about contraceptive methods

**Figure 1:**
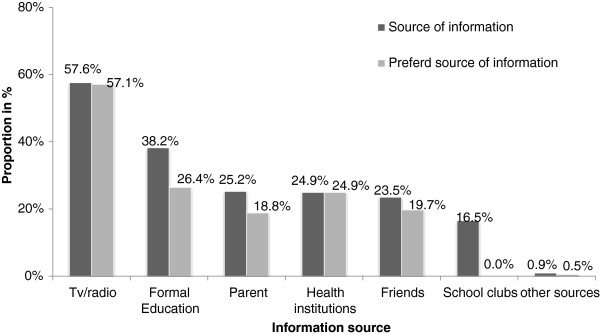
**Main sources of information on methods of contraception among secondary school female students in Mekelle town, northern Ethiopia, 2012.**

Among study participants with awareness on contraceptive methods, more than half (57.6%) reported that they received their information from TV/radio, followed by formal education (38.2%). With regard to the choice of their information source, 57.1% and 26.4% of the participants preferred TV/radio and formal education, respectively (Figure 1).

### Discussion about sexual and reproductive health issues with parents and peers

**Figure 2:**
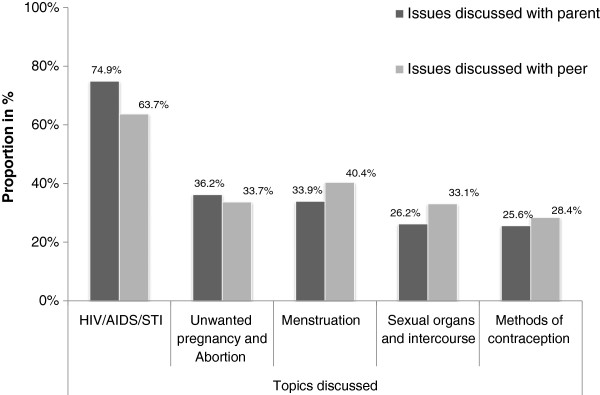
**Sexual and reproductive health issues discussed with parent and peer among secondary school female students in Mekelle town, northern Ethiopia, 2012.**

In the past six months, 351(43.5%) of the participants reportedly discussed SRH matters with their parent/s. A larger proportion, 493(61.1%), of the students had discussed SRH matters with their peer/s during the same period. A lower proportion of students, 25.6%, and 28.4% reported to have discussed methods of contraception with parent/s and peer/s, respectively (Figure 2).

Compared to younger age groups, a higher proportion, 156(70.3%), of 18–21 years age group students, reportedly discussed SRH issues with their peers. Of those students whose mothers were illiterate, 196(82.8%) reported having discussed SRH issues with their peers (Table 4).

**Table 4:** **Socio**-**demographic**, **educational and parental characteristics by discussion on SRH issues with parents and peers during the last 6 months**, **among female students**, **Mekelle**, **north Ethiopia**, **2012**

**Characteristics**	**Discussed on SRH issues with parent**		**Pearson Chi2**	**Discussed on SRH issues with peers**		**Pearson Chi2**
	**Yes**	**No**	**p**-**value**	**Yes**	**No**	**p**-**value**
**Respondents’ age in years**			0.132			0.003
13-15	70(37.2%)	118(62.8%)		104(55.3%)	84(44.7%)	
16-17	178(44.8%)	219(55.2%)		233(58.7%)	164(41.3%)	
18-21	103(46.4%)	119(53.6%)		156(70.3%)	66(29.7%)	
**Respondents’ religion**			0.127			0.530
Orthodox	306(44.3%)	384(55.7%)		427(61.9%)	263(38.1%)	
Muslim	21(31.8%)	45(68.2%)		37(56.1%)	29(43.9%)	
Other	24(47.1%)	27(52.9%)		29(56.9%)	22(43.1%)	
**Respondents’ origin of residence area**			0.701			0.188
Urban	55(45.1%)	67(54.9%)		68(55.7%)	54(44.3%)	
Rural	296(43.2%)	389(56.8%)		425(62.0%)	260(38.0%)	
**Respondents’ educational level**			0.167			< 0.001
Junior school (grade 9th and 10th)	190(41.4%)	269(58.6%)		245(53.4%)	214(46.6%)	
Senior school (grade 11th and 12nd)	161(46.3%)	187(53.7%)		248(71.3%)	100(28.7%)	
**Stream of study (n** = **348)**			0.233			0.638
Social sciences	35(40.7%)	51(59.3%)		63(73.3%)	23(26.7%)	
Natural sciences	126(48.1%)	136(51.9%)		185(70.6%)	77(29.4%)	
**School attending**			0.113			0.121
Public	310(44.6%)	385(55.4%)		432(62.2%)	263(47.8%)	
Private	41(36.6%)	71(63.4%)		61(54.5%)	51(45.5%)	
**Fathers’ level of education**			0.269			0.064
No education	94(39.3%)	145(60.7%)		146(61.1%)	93 (38.9%)	
Primary education	66(41.5%)	93(58.5%)		88 (55.3%)	71(44.7%)	
Secondary education	97(48.0%)	105(52.0%)		138 (68.3%)	64(31.7%)	
Tertiary education	94(45.4%)	113(54.6%)		121(58.5%)	86(41.5%)	
**Mothers’ level of education**			0.685			0.046
No education	128(41.0%)	184(59.0%)		196(82.8%)	116(17.3%)	
Primary education	98(43.9%)	125(56.1%)		137(61.4%)	86(38.6%)	
Secondary education	65(46.4%)	75(53.6%)		93(66.4%)	47(33.6%)	
Tertiary education	60(45.5%)	72(54.5%)		67(50.8%)	65(49.2%)	

### Factors affecting awareness on contraceptive methods

In bivariate logistic regression, age, educational level, discussion on SRH issues with parents and peers, attending school mini-media, and ever had sexual partner were predictors of students’ awareness on methods of contraception.

However, in multivariate logistic regression, only discussion on SRH issues with parents [(AOR =2.56(95% CI: 1.45, 4.50)] and peers [(AOR = 2.46(95% CI: 1.50, 4.03)] was found to be significantly associated with students’ awareness on methods of contraception. In contrast, type of school (public or private), stream of study (natural or social), origin of residence (urban or rural), parental education, and perceived economic status had no statistically significant association with contraception awareness (Table 5).

**Table 5:** **Factors associated with awareness of secondary school female students on methods of contraception**, **Mekelle town**, **northern Ethiopia**, **2012**

**Characteristics**	**Had awareness**				
	**Yes**	**No**	**COR (95% CI)**	**AOR (95% CI)**	**p**-**value**
**Age in years**					
13-15	163	25	1	1	
16-17	345	52	1.02(0.61, 1.70)	0.76(0.44, 1.33)	0.339
18-21	208	14	2.28(1.15, 4.52)	1.00(0.41, 2.46)	0.999
**Educational level**					
Junior school (grade 9th & 10th)	391	68	1	1	
Senior school (grade 11th & 12nd)	325	23	2.46(1.50, 4.03)	1.90(0.98, 3.67)	0.057
**Discussion on SRH issues with parent/s**					
Yes	333	18	3.53(2.06, 6.03)	2.56(1.45,4.50)	0.001
No	383	73	1	1	
**Discussion on SRH issues with peer/s**					
Yes	463	30	3.72(2.34, 5.91)	2.46(1.50,4.03)	<0.001
No	253	61	1	1	
**Listen school mini media**					
Yes	411	38	1.88(1.21, 2.93)	1.48(0.94, 2.36)	0.094
No	305	53	1	1	
**Ever had sexual partner**					
Yes	159	12	1.88(1.00, 3.54)	1.17(0.60,2.29)	0.647
No	557	79	1	1	

## Discussion

The current study is one of few studies in Ethiopia to examine the effect of parent and peers communication on contraceptive awareness among secondary school students. Our study has excluded married women since the main purpose of the study was to examine the effect of parent and peers communication on contraceptive awareness among in-school unmarried youths, on the basis that awareness of married youths on contraceptive methods is very high [8].

According to this study, about 127(15.8%) of the students had ever had sexual intercourse. The figure is higher compared with a study done with high school female students in Bullen woreda (northwest Ethiopia), where 3.9% had reportedly been sexually active [34]. This might be due to the cultural inhibition both to have premarital sex and to report having had sex in northwest Ethiopia. However, the figure is much lower than a study undertaken in Zeway high school (south central Ethiopia) where 31.5% reported being sexually experienced [31]. The higher figure in this study might be due to the fact that the study subjects were males who tend to report more sexual activity in surveys than females.

On the other hand, studies among undergraduate university female students show a higher proportion of sexually active students than in our study, ranging from university-based respondents in Bahir Dar and Gondar (16.5%) [35], Addis Ababa (23.4%) [36], Kenya (47.6%) [37] and Ghana (38%) [38]. This could be because female students in these universities were away from family and were also older than the secondary school students in our study.

One-fifth (21.2%) of the students reported having had just one sexual partner in their life. This figure is much lower than a study conducted in Bahir Dar and Gondar universities (37.9%) [35], and might be due to the difference in age and place of residence, where university students are living away from their families and are therefore exposed to more partner opportunities than high school students who still live at home.

Although the unintended pregnancy rate of 15.7% in this study is comparable with a study among university students in Bahir Dar and Gondar Universities (16.5%) [35], there was a much lower rate (3.5%) [36] of unintended pregnancy among female students attending Addis Ababa University in spite of their being more sexually active. This could be due to university students possibly having better knowledge about how to protect against pregnancy as well as better awareness about contraceptive methods. In our study, we have observed a higher induced abortion rate than the national average of 2.3% [39-41], but a lower rate (14.2%) than found in a study in Bahir Dar and Gondar universities (15.5%) [35]. Other Ethiopian studies have found the mean age of women who seek abortion to be 23 years, of whom the majority (54%) were single [39-41]. This is broadly consistent with the findings of our study, whose subjects were unmarried young female students.

This study revealed that the majority, 88.7%, of the students have ever heard about methods of contraception, mentioning at least one contraceptive method. A further analysis of Demographic and Health Survey (DHS) of 2005 result in Ethiopia showed that a slightly lower proportion, three of every four people aged 15–24 years old, knew of at least one contraceptive method. This might be because our study population constituted an educated group, while in the DHS survey both educated and non-educated youths were included. This finding is also slightly lower than the previous studies report from Addis Ababa university female students, where 91.3% were knowledgeable about contraceptive methods [36]. This could be explained by the fact that students in tertiary education are more likely to be sexually active and to have sex and reproductive health related information than secondary school students.

The male condom was the most frequently mentioned method of contraception (66.3%) reported by our respondents. This may be due to the fact that the condom is promoted not only as a contraceptive method but also for protecting against STI including HIV/AIDS.

Awareness of the Emergency Oral Contraceptive (EOC) was much lower (39.4%) among the participants of this study than in other similar studies. For example, in a study conducted in 2012 among 368 undergraduate female students of Addis Ababa University showed that 84.2% were aware of EOC [36], while a study from university students in northwest Ethiopia reported awareness at 67.1% of the respondents [35]. This variation might be due to differences in their level of education and the greater promotion and availability of EOC in tertiary than in secondary schools.

In the current study, 82(64.6%) of those who had ever had sex reported that they used some kind of contraceptive during their last sexual intercourse. In a study among 368 sexually active adolescents in Cape Verde (West Africa), a slightly higher (69.3%) proportion reported using a contraceptive in their last sexual intercourse [42]. This could be due to lower awareness, and poorer access to contraceptive methods among our study participants.

Male condom and withdrawal methods were the most commonly used type of methods among sexually active students in the current study, the Cape Verde study [42], as well as in another study conducted in Uganda [24]. On the other hand, a study among reproductive health service users in Haiti showed that fewer adolescents (42%) used a contraceptive during their last sexual intercourse [43]. This could have been because their first sexual partner was older, which can lead to power differentials between the partners [43], which in turn can affect the decision making process to use contraceptives.

In the current study and other previous studies, the main source of information about contraceptive methods was the media, in the form of TV and radio. In our study, more than half (57.6%) received their information from media, as compared to a study in Addis Ababa university, where the proportion was 75.5% [36]. This difference might have arisen because the media can be accessed by more students in the university compared to high school students, regardless of their parental socioeconomic status.

However, in contrast to our study where media (TV/radio) were the main source of information for SRH issues, a study among high school students in northwest Ethiopia showed that the most frequently mentioned source of information was school (83.3%) [34]. This difference could be because of low access to TV and radio by students in this more remote area, and further, even for those who had access, the media material may not include tailored and targeted SRH programs for that region. A study among undergraduate students indicated that the main source of information for contraceptive was the media, which can be accessed by most students in urban areas [36].

In the previous six months, fewer than half (43.5%) of the participants had discussed SRH matters with their parent/s. Other studies in this issue have indicated that a smaller proportion, 32.4% [33] and 28.9% [34], of students had discussion with their parents on SRH issues. There are no previous studies which reported on associations between parent and peer communications and awareness of contraceptive methods, but we found that 94.9% of the students who discussed SRH issues with their parents in this study were aware of contraception methods, which was significantly more than those who had not discussed SRH issues with their parents. Similarly, 493(61.1%) of the participants reported having had discussion on SRH matters with their peer/s in the past six months, a finding which is comparable with other study in eastern Wollega Zone, west Ethiopia (59.5%)[33]. Additionally, study participants who have discussed on SRH issues with their peers (93.9%) were more likely to have awareness on contraceptive methods than their counter parts (84%). These findings are supported by a South African study, which pointed to the potential importance of parent and peer communication as a means of creating awareness about SRH issues [30].

About three quarters (74.9%) of the study participants reported to have discussed sexually transmitted infections including HIV/AIDS with their parent/s, which is similar to studies conducted in other part of Ethiopia, 67.8% [33] and 78.6% [34]. Similarly, about two thirds (63.7%) of the students had discussed the topic with their peer/s. Another study conducted in Benishangul Gumuz region, northwest Ethiopia, reported that almost three quarters (73.1%) of the participants had discussed the topic with their peers [34]. These relatively high proportions may be the result of concern and worry about HIV and AIDS caused by the topic being commonly discussed in the media. By contrast, just 25.6%, of the students had discussed methods of contraception with their parent/s, although this was higher than in another study in west Ethiopia, where only 10% had done so [33].

Students who were enrolled in higher (senior secondary schools) educational level (93.4%) were more likely to have awareness on contraceptive methods compared to those who were in junior schools (85.2%). This could be due to youths in higher educational level being more likely to engage in discussion with their parents about SRH issues compared to their counter parts [33], and these groups are more likely to have better awareness on contraceptive methods, which is also indicated as main finding in our study.

In this study, school attendance (public or private), students’ stream of study (natural or social), origin of residence (urban or rural) and parental educational level, and perceived economic status were not significantly associated with female secondary students’ awareness of contraceptive methods in northern Ethiopia. This could be due to most of the students’ sources of information about contraceptive methods being national/local audio-visual media, which have considerable amounts of programming related to contraceptive methods tailored for young people. In addition to this, media could be easily accessed in urban areas such as our study setting.

Three limitations of the study should be mentioned. First, despite the efforts made to ensure data validity, it is not possible to guarantee that students provided honest answers to all the questions, especially those concerned with sensitive issues such as sex. Second, the study was conducted in six selected urban-based secondary school female students in the northern part of Ethiopia, and thus the findings cannot be generalized for all secondary school female students in Ethiopia. Third, male students were not included in the study.

## Conclusions

This study showed that many of our female student respondents had a history of sexual intercourse, though significant numbers of students had no awareness about how to prevent pregnancy. Six months prior to the study, fewer than half of the participants reported to have discussed SRH matters with their parent/s, but almost two-thirds of the students had discussed SRH matters with their peer/s. Although audio-visual media such as television and radio were participants’ main and preferred source of information about contraceptive methods, we found that discussing SRH issues with parents and peers positively affected students’ awareness about contraceptive methods. Therefore, strategies to enhance and improve open communication on sexual and reproductive health between parents and students, as well as peer-to-peer education in schools, should be developed and strengthened as a means of increasing awareness about contraceptive methods. In a community where some young people feel shame when talking about SRH issues with families and even with their peers, such programmes could play an important role in helping teenage girls and young women to prevent unwanted pregnancy and its consequences.

## Abbreviations

AIDS: Acquired immunodeficiency syndrome; AOR: Adjusted odds ratio; COR: Crude ddds ratio; EOC: Emergency oral contraceptives; HIV: Human immunodeficiency of virus; IUD: Intrauterine device; LAM: Lactation amenorrhea method; SPSS: Statistical package for social sciences; SRH: Sexual and reproductive health; STIs: Sexually transmitted infections; TV: Television; OR: Odds ratio.

## Competing interests

The authors declare that they have no competing interests.

## Authors’ contributions

YAM was involved in study conception, data processing, and data analysis, interpretation of the results and drafting of the manuscript. YB participated in conception and review of the study. HLR and JK helped in reviewing the manuscript. All authors read and approved the final manuscript.

## Pre-publication history

The pre-publication history for this paper can be accessed here:

http://www.biomedcentral.com/1471-2458/14/252/prepub

## References

[B1] UNDO/UNFPA/WHO Special program of Research Development and Research Training in Human Reproductive Health (HRP), Progress in Reproductive Health Research 2003 Geneva, Switzerland: World Bank. No 64

[B2] WHO Strengthening the Provision of Adolescents- Friendly Health Service to meet the health and development needs of adolescents in Africa 2000 Harare, Zimbabwe: A consensus statement 17 21

[B3] United Nations (UN) World population prospects 2006 New York: The 2006 revision

[B4] Elster AB Kznets NJ American Guidelines for Adolescent Preventing Services (GAPS), Recommendation and Rationale 2000 Philadelphia: Lippincott Williams & Wilkins

[B5] Josaphat K Florence M Elisabeth F Krstina G Emergency contraception and fertility awareness among University students in Kampala, Uganda African Health Sci 2006 6 4 194 200 10.5555/afhs.2006.6.4.194 PMC1832063 17604507

[B6] WHO Research on Reproductive Health-Biennial Reports 2000–2001 Geneva

[B7] Mangiaterra V Pendse R Mclure K Rosen J Adolescent pregnancy Department of making pregnancy safer (MPS) 2008 WHO MPS note http://www.who.int/maternal_child_adolescent/documents/mpsnnotes_2_lr.pdf

[B8] Central Statistical Agency [Ethiopia] and ORC Macro Ethiopia Demographic and Health Survey 2011 2012 Addis Ababa, Ethiopia and Calverton, Maryland, USA

[B9] WHO, Gutmacher Institute Facts on induced abortion worldwide 2007 http://www.who.int/reproductivehealth/publications/unsafe_abortion/induced_abortion_2012.pdf

[B10] Darroch JE Singh S Frost JJ Differences in teenage pregnancy rates among five developed countries: the roles of sexual activity and contraceptive use Fam Plann Perspect 2001 33 244 50 10.2307/3030191 11804433

[B11] Singh S Bankole A Woog V Evaluating the need for sex education in developing countries: sexual behavior, knowledge of preventing sexually transmitted infections/HIV and unplanned pregnancy Sex Educ 2005 5 307 31 10.1080/14681810500278089

[B12] Kaufman CE De Wet T Stadler J Adolescent pregnancy and parenthood in South Africa Stud Fam Plann 2001 32 147 60 10.1111/j.1728-4465.2001.00147.x 11449863

[B13] Center for communication John Hopkins School of Public Health USA Meeting the needs of young adults 1995 Baltimore, Maryland: Population Information Program; Population report No 3

[B14] Leon S Philip D Contraception in USA A clinical guide for contraception 1996 Philadelphia: Lippincott Williams & Wilkins Second edition No 1

[B15] Center for communication John Hopkins School of public health USA: Low dose pill. Population report No 3 1988 Baltimore, Maryland: Population Information Program

[B16] Center for communication John Hopkins School of public health 1999 Baltimore, Maryland, USA: Why family planning matters Population Information Program; Population report No 2

[B17] Linda H Renee E Jane F Vinit S Global perspectives on the sexual and reproductive health of adolescents: patterns, prevention, and potential Lancet 2007 369 1220 31 10.1016/S0140-6736(07)60367-5 17416266

[B18] Central Statistical Agency [Ethiopia] and ORC Macro Ethiopia Demographic and Health Survey 2005 2006 Addis Ababa, Ethiopia and Calverton, Maryland, USA

[B19] Barbara S Monica J Grant A Blane K The changing context of sexual initiation in Sub-Saharan Africahttp://www.popcouncil.org/uploads/pdfs/wp/206.pdf

[B20] Kalamuss D Davidson A Cohall A Laraque D Cassell C *Preventing sexual risk behaviors and pregnancy among Teenagers*: *Linking Research and Programs* Perspect Sex Reprod Health 2003 35 2 87 93 10.1363/3508703 12729138

[B21] United Nations: program on HIV/AIDS report of the global AIDS epidemic 2004 2004 New York: United Nations

[B22] Billy JOG Landale NS Grady WR Zimmerle DM Effects of sexual activity on adolescent social and psychological development Soc Psychol Q 1988 51 190 212 10.2307/2786919

[B23] Oni T Prinsloo E Nortje J Joubert G High school students’ attitudes, practice and knowledge of contraception in Jozini, Kwazulu-Natal SA Fam Pract 2005 47 6 54 57

[B24] Josaphat K Florence M Elisabeth F Kristina G Emergency contraception and fertility awareness among university students in Kampala. Uganda. Karolinska Institute Dept. of woman and child health division of obstetrics and gynecology African Health Sci 2006 6 4 194 200 10.5555/afhs.2006.6.4.194 PMC1832063 17604507

[B25] Adetokunbo T Oluwarotimi A Abiola B Adeniyi A Dele O Lukeman S Contraception knowledge and usage amongst female secondary school students in Lagos, southwest Nigeria J Public Health Epidemiol January 2011 39 1 34 37

[B26] Govindasamy P Aklilu K Hailom B Youth Reproductive Health in Ethiopia 2002 Calverton, Maryland: ORC MACRO http://www.studymode.com/essays/Youth-Reproductive-Health-In-Ethiopia-1072788.html

[B27] Fantahun M Chala F Loha M Knowledge, attitude and practice of family planning among senior high school students in North Gondar Ethiop Med J 1995 33 21 9 7895743

[B28] Jerman P Constantine NA Demographic and psychological predictors of parent-Adolescent communication about sex: a representative statewide analysis 2010 J Youth Adolesc 2010 39 1164 1174 10.1007/s10964-010-9546-1 20458614 PMC2917005

[B29] Kelly Ladin L’E Jackso C Socialization influences on early adolescents’ cognitive susceptibility and transition to sexual intercourse J of rese on Adolescence 2008 18 2 353 378 10.1111/j.1532-7795.2008.00563.x

[B30] Hallman K Socio-economic Disadvantage and Unsafe sexual behaviors among young men and women in South Africa. Population council, Population Research Division 190 2004 New Work

[B31] Teffa N Bjune G Sundby J Gaustad P Alestrom A Prevalence of Gonococcal and chlamidial infections and sexual risk behaviors among youth in Addis Ababa, Ethiopia Sex transm Dis 2002 29 12 828 833 10.1097/00007435-200212000-00015 12466727

[B32] Taffa N Haimanot R Desaleng S Tesfaye A Mohammed K Do parents and young people communicate on sexual matters? The situation of life education (FLE) in rural town of Ethiopia Ethiop J Health Dev 1999 15 2 109 116

[B33] Tesso W Fantahun A Enquselassie F Parent-young people communication about sexual and reproductive health in E/Wollega Zone. West Ethiopia: implication for interventions BMC Reproductive Health 2012 9 13 10.1186/1742-4755-9-13 PMC3495660 22898627

[B34] Gebre D Fantahun M Assessing communication on sexual and reproductive health issues among high school students with their parents, BullenWoreda, Benishangul Gumuz Region, North West Ethiopia Ethiop J Health Dev 2010 24 2 90 94

[B35] Wassie B Belyhun Y Moges B Amare B Effect of emergency oral contraceptive use on condom utilization and sexual risk taking behaviors among university students. Northwest Ethiopia: a cross sectional study BMC Res Note 2012 5 501 10.1186/1756-0500-5-501 PMC3494538 22971668

[B36] Ahmed Moussa KM Petterson KO Asamoah BO Assessing knowledge, attitude, and practice of emergency contraception: a cross-sectional study among Ethiopian undergraduate female students BMC public Health 2012 12 110 10.1186/1471-2458-12-110 22321964 PMC3293041

[B37] Marry BA Mike M Sexual risk behavior among Kenyan University students J Arizona-Nevada Academy 2007 39 2 91 98 10.2181/036.039.0205

[B38] El-Adas A The Resolution of Unintended Pregnancy among Female Students at the University of Ghana, Legon 2007 Regional Institute for Population Studies: University of Ghana, Legon

[B39] Singh S Fetters T Gebreselassie H Abdella A Gebrehiwot Y Kumbi S Audam S The estimated incidence of induced abortion in Ethiopia IntPerspect on Sexual and Rep Health 2010 36 1 16 25 10.1363/3601610 20403802

[B40] Gebreselassie H Fetters T Singh S Abdella A Gebrehiwot Y Tesfaye S Geressu T Kumbi S Caring for women with abortion complications in Ethiopia: National estimates and future implications Int Perspect Sex Reprod Health 2010 36 1 6 15 10.1363/3600610 20403801

[B41] Nasir T Pharm B Knowledge, attitude, and practice of emergency contraception among graduating female students of Jimma University, southwest Ethiopia Ethiop J Health Dev 2010 20 91 97 PMC3275837 22434966

[B42] Mendes C Valenti EV Kanikadan Y Carlos L Condom use at last sexual relationship among adolescents of Santiago Island Cape Verde, West Africa BMC Reproductive Health 2012 9 29 10.1186/1742-4755-9-29 PMC3538512 23153259

[B43] Gomez A Speizer I Reynolds H Murray N Beauvais H Age difference at sexual debut and subsequent reproductive health: is there a link? BMC Reproductive Health 2008 5 8 10.1186/1742-4755-5-8 PMC2585071 18976477

